# Salivary Antimicrobial Peptide Histatin-5 Does Not Display Zn(II)-Dependent or -Independent Activity against Streptococci

**DOI:** 10.1021/acsinfecdis.2c00578

**Published:** 2023-02-24

**Authors:** Louisa J. Stewart, YoungJin Hong, Isabel R. Holmes, Samantha J. Firth, Yasmin Ahmed, Janet Quinn, Yazmin Santos, Steven L. Cobb, Nicholas S. Jakubovics, Karrera Y. Djoko

**Affiliations:** † Department of Biosciences, Durham University, Durham DH1 3LE, United Kingdom; ‡ Biosciences Institute, Newcastle University, Newcastle NE2 4HH, United Kingdom; § Department of Chemistry, Durham University, Durham DH1 3LE, United Kingdom; ∥ School of Dental Sciences, Newcastle University, Newcastle NE2 4BW, United Kingdom

**Keywords:** antimicrobial peptide, histatin, zinc, nutritional immunity, *Streptococcus*

## Abstract

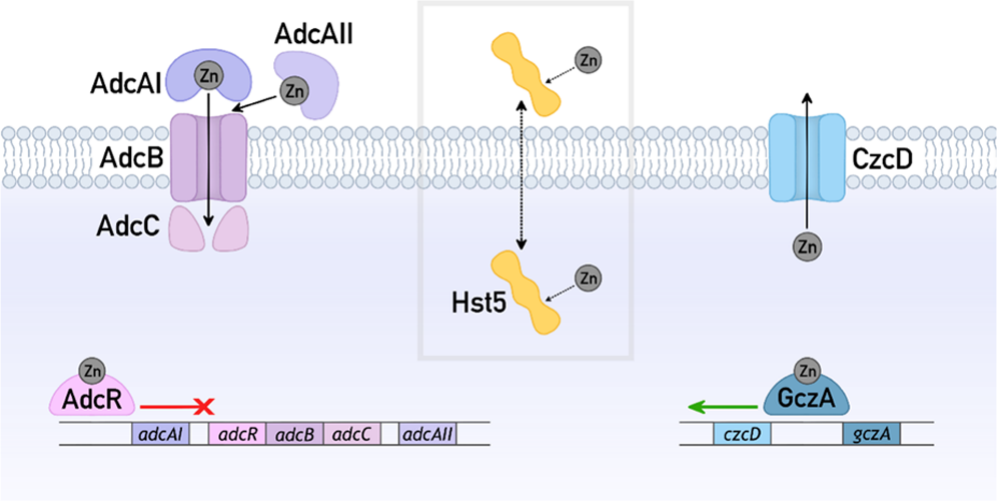

Histatin-5 (Hst5) is a member of the histatin superfamily of cationic, His-rich, Zn(II)-binding peptides in human saliva. Hst5 displays antimicrobial activity against fungal and bacterial pathogens, often in a Zn(II)-dependent manner. In contrast, here we showed that under *in vitro* conditions that are characteristic of human saliva, Hst5 does not kill seven streptococcal species that normally colonize the human oral cavity and oropharynx. We further showed that Zn(II) does not influence this outcome. We then hypothesized that Hst5 exerts more subtle effects on streptococci by modulating Zn(II) availability. We initially proposed that Hst5 contributes to nutritional immunity by limiting nutrient Zn(II) availability and promoting bacterial Zn(II) starvation. By examining the interactions between Hst5 and *Streptococcus pyogenes* as a model *Streptococcus* species, we showed that Hst5 does not influence the expression of Zn(II) uptake genes. In addition, Hst5 did not suppress growth of a Δ*adcAI* mutant strain that is impaired in Zn(II) uptake. These observations establish that Hst5 does not promote Zn(II) starvation. Biochemical examination of purified peptides further confirmed that Hst5 binds Zn(II) with high micromolar affinities and does not compete with the AdcAI high-affinity Zn(II) uptake protein for binding nutrient Zn(II). Instead, we showed that Hst5 weakly limits the availability of *excess* Zn(II) and suppresses Zn(II) toxicity to a Δ*czcD* mutant strain that is impaired in Zn(II) efflux. Altogether, our findings led us to reconsider the function of Hst5 as a salivary antimicrobial agent and the role of Zn(II) in Hst5 function.

Antimicrobial peptides are short, often cationic peptides that are secreted by diverse organisms from across the domains of life.^1^ These peptides usually act as immune effectors that kill invading microbes as part of the host innate immune system, but many also play key functions in the normal biology of the host organism. A subfamily of antimicrobial peptides binds metals. Some of these metallo-peptides become activated upon metal binding,^2−4^ for instance, by folding into an optimal conformation for disrupting microbial membranes or for acting on their targets (*e.g*., clavanin A from tunicates^4^ and piscidin from fish^2^). Other metallo-peptides bind metals and withhold these essential nutrients away from microbes, causing them to starve (*e.g*., microplusin from cattle ticks^5^).

Histatins comprise a family of cationic, His-rich, metallo-peptides in the saliva and tears of humans and some higher primates.^6−8^ These peptides are derived from two parent peptides, namely, Histatin-1 and Histatin-3.^6,9^ Both parent histatins are expressed by the salivary and tear glands.^10,11^ Upon secretion in saliva into the oral cavity, the parent histatins are rapidly processed into shorter fragments^12−14^ by unidentified human salivary proteases or proteases from resident oral microbes. Whether the parent histatins are proteolytically degraded in tears is currently unknown. Of the various salivary fragments, Histatin-5 (Hst5; Table 1) is the best characterized *in vitro*.

**Table 1:**
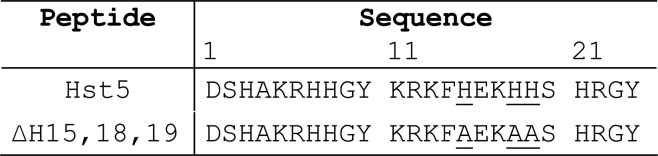
Hst5 Peptides Used in This Work

Hst5 is noted for its ability to kill the fungus *Candida albicans*(15,16), and several pathogenic bacterial species, namely, *Pseudomonas aeruginosa*, *Staphylococcus aureus*, *Acinetobacter baumanii*, *Enterococcus faecium*, and *Enterobacter cloacae*.^16^ Unlike other antimicrobial peptides, Hst5 does not appear to permeabilize fungal membranes, although it does destabilize some bacterial membranes.^16^ Beyond its direct action on membranes, the antimicrobial activity of Hst5 requires the peptide to be internalized into the cytoplasm, usually *via* energy-dependent pathways for peptide uptake.^16,17^ Once in the cytoplasm, Hst5 is thought to encounter its targets, which in *C. albicans* include the mitochondria^18^ but in bacteria remain unidentified, and causes toxicity *via* multiple pathways that are not fully elucidated.^15,18^

Hst5 contains a characteristic Zn(II)-binding motif, His-Glu-x-His-His (Table 1), but whether Hst5 associates with Zn(II) in saliva is unknown. Likewise, whether Zn(II) binding is essential for the antimicrobial activity of Hst5 is unclear. Synthetic Hst5 derivatives that lack one or all three putative Zn(II)-binding His residues remain active against *C. albicans*.^19^ In addition, conflicting reports show that addition of Zn(II) can both enhance^20^ and suppress^21^ Hst5 activity against this fungus. However, a recent report indicates that the role of Zn(II) is concentration-dependent: low concentrations of added Zn(II) enhance the antimicrobial activity of Hst5 against *C. albicans* (compared with the control without any added Zn(II)), while high concentrations of added Zn(II) suppress it.^22^

Beyond histatins and Zn(II)-binding metallo-peptides, Zn(II)-dependent host innate immune responses are well described. In response to microbial infection, Zn(II) levels and those of Zn(II)-binding or Zn(II)-transporting proteins within a host organism can rise and fall, leading to fluctuations in Zn(II) availability within different niches in the infected host. Increases in Zn(II) availability promote microbial poisoning while decreases in Zn(II) availability promote microbial starvation. These antagonistic host responses, known as “nutritional immunity”,^23^ suppress microbial growth in the host and inhibit the progress of infectious disease. Although Zn(II) influences the activity of Hst5,^22^ it is unclear whether histatins themselves participate in nutritional immunity by modulating Zn(II) availability to microbes.

The healthy human oral cavity and oropharynx are colonized by a mixture of microbial species, with *Streptococcus* as the most abundant taxon.^24−28^ Some species, such as *S. gordonii* and *S. sanguinis*, are considered commensals. These species contribute to oral health, for example, by inhibiting colonization by competitor species.^29,30^ Some streptococcal species are considered pathogenic. For example, *S. mutans* and *S. pyogenes* are associated with dental caries and pharyngitis,^31^ respectively. Nevertheless, asymptomatic carriage of these pathogenic species is common^32^ and these species are generally considered normal components of the healthy oral and oropharyngeal microflora. Importantly, all streptococci are opportunistic pathogens that can cause disseminated infections, such as bacterial infective endocarditis.^33^

The goals of this study were to determine the antibacterial activity of Hst5 against oral and oropharyngeal streptococci, and to investigate the potential role of this peptide in influencing Zn(II) availability to the streptococci as a component of nutritional immunity. Based on the established features of nutritional immunity, we specifically examined whether Hst5 limits Zn(II) availability (and promotes microbial Zn(II) starvation) and/or raises Zn(II) availability (and promotes Zn(II) poisoning).

## Results

### Hst5 Does Not Kill Oral or Oropharyngeal Streptococci

**Figure 1:**
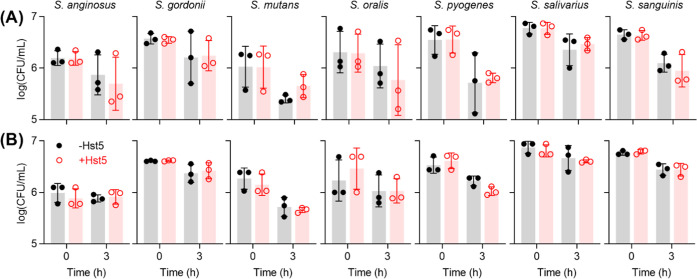
Effects of Hst5 on survival of streptococci in (A) phosphate buffer and (B) artificial saliva buffer. Bacteria were incubated in phosphate buffer (10 mM, pH 7.4; *N* = 3) or artificial saliva buffer (pH 7.2–7.4; *N* = 3), with (○) or without (●) Hst5 (50 μM), and sampled at *t* = 0 and 3 h for enumeration. Hst5 did not affect the survival of any species in either buffer (*P* = 0.73, 0.99, 0.57, 0.72, 0.85, 0.71, 0.50 in phosphate buffer, and 0.72, 0.71, 0.43, 0.56, 0.52, 0.48, and 0.86 in artificial saliva buffer, for *S. anginosus*, *S. gordonii*, *S. mutans*, *S. oralis*, *S. pyogenes*, *S. salivarius*, and *S. sanguinis*, respectively).

There is little consensus regarding the antibacterial activity of Hst5 against streptococci—it varies depending on the species or experimental conditions,^34−40^ but the chemical and molecular reasons for these discrepancies have not been identified. In this work, the ability of Hst5 to kill seven oral or oropharyngeal streptococci, namely, *S. anginosus*, *S. gordonii*, *S. mutans*, *S. oralis*, *S. pyogenes*, *S. salivarius*, and *S. sanguinis*, was examined in parallel. Following the approach used previously for *C. albicans* and ESKAPE pathogens, these kill assays were performed for several hours in dilute phosphate buffer (10 mM).^16,20^ Under these conditions, up to 50 μM Hst5 (*ca.* total histatin concentrations in fresh salivary secretions^13^) did not promote killing of the streptococcal species (Figure 1A), even when the assay was extended to 24 h (Figure S1). Consistent with a previous report,^16^ parallel control experiments showed that Hst5 killed *P. aeruginosa* and *C. albicans* (Figure S2), confirming that our peptide preparations were active.

Like other cationic antimicrobial peptides, the antimicrobial activity of Hst5 is influenced by pH and ionic strength.^16,19,41−45^ To better reflect the physiological context in which Hst5 plays a role, the kill assays were repeated in an artificial, synthetic “saliva buffer”, whose pH and ionic composition approximate that of saliva (Table S1A). Again, Hst5 did not kill any of the streptococci (Figures 1B and S1). Interestingly, under these new conditions, Hst5 did not kill the control organisms *P. aeruginosa* and *C. albicans* (Figure S2). The high ionic strength of the saliva buffer likely interferes with electrostatic binding of the peptide to surface proteins or membranes of these control organisms,^16,46^ and subsequent internalization and killing. To better understand the activity of Hst5 under conditions that are more characteristic of saliva, further kill assays below used the artificial saliva buffer.

### Zn(II) Does Not Influence the Activity of Hst5 against Streptococci

**Figure 2:**
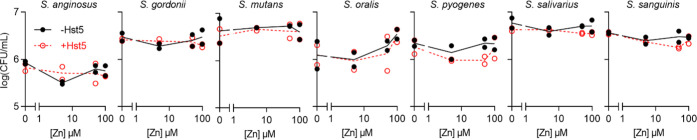
Effects of Zn(II) and Hst5 on survival of streptococci in artificial saliva buffer. Bacteria (*N* = 2) were incubated in artificial saliva buffer in the presence of added Zn(II) (0, 5, 50, or 100 μM), with (○) or without (●) Hst5 (50 μM), and sampled at *t* = 3 h for enumeration. Addition of Zn(II) did not influence the effects of Hst5 on the survival of any species (*P* values for the interaction between Zn(II) and Hst5 = 0.40, 0.46, 0.96, 0.98, 0.69, 0.45, and 0.09 for *S. anginosus*, *S. gordonii*, *S. mutans*, *S. oralis*, *S. pyogenes*, *S. salivarius*, and *S. sanguinis*, respectively).

Saliva typically contains low micromolar levels of total Zn(II) (between 0.2 and 3 μM have been reported^47^), although the speciation or bioavailability of this metal ion is poorly defined. Our artificial saliva buffer is Zn(II)-deplete (low nanomolar concentrations of Zn(II) are routinely detected by inductively coupled plasma mass spectrometry (ICP MS)). Thus, to determine if the activity of Hst5 against streptococci is Zn(II)-dependent, the kill assays were repeated in the presence of added Zn(II). The results showed that added Zn(II), whether substoichiometric (5 μM), stoichiometric (50 μM), or super-stoichiometric (100 μM) relative to Hst5 (50 μM), neither suppresses nor enhances killing of the seven streptococcal species by Hst5 (Figure 2).

### Hst5 Does Not Contribute to Zn(II)-Dependent Nutritional Immunity

**Figure 3:**
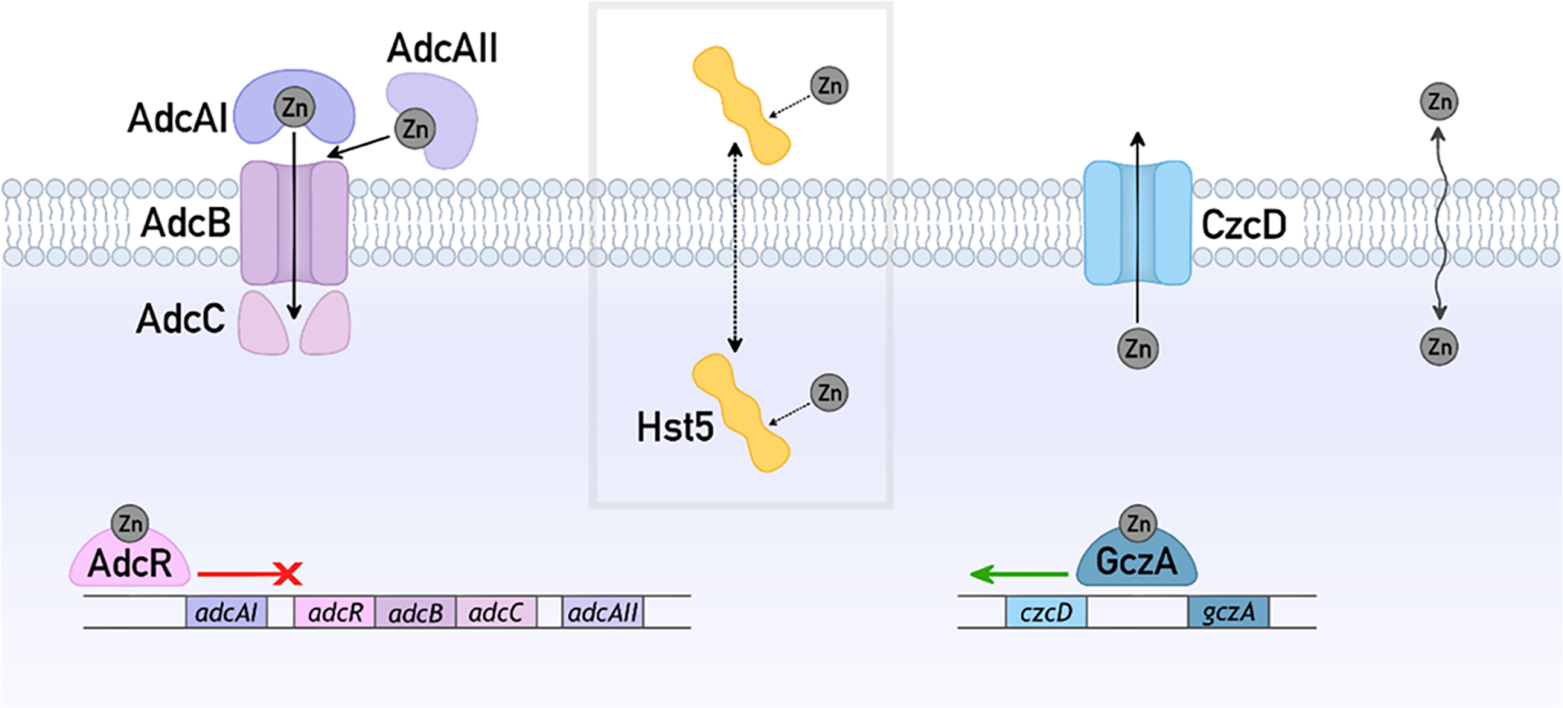
Zn(II) homeostasis in GAS and hypothesized actions of Hst5. Zn(II) uptake: AdcAI and AdcAII capture extracellular Zn(II) and transfer this metal to AdcBC for import into the cytoplasm. These proteins are transcriptionally upregulated in response to decreases in Zn(II) availability and Zn(II) starvation (and downregulated in response to increases in Zn(II) availability).^49^ Alternatively, Zn(II) may enter the cytoplasm *via* nonspecific cation transporters (wavy arrow). Zn(II) efflux: CzcD exports excess Zn(II) out of the cytoplasm. It is transcriptionally upregulated by GczA in response to increases in Zn(II) availability and Zn(II) poisoning.^50^ Alternatively, Zn(II) may exit the cytoplasm *via* nonspecific cation transporters (wavy arrow). Hypothesized actions of Hst5: Hst5 may bind extracellular Zn(II) and either remain extracellular to suppress Zn(II) availability or become internalized as the Zn(II)–Hst5 complex and increase Zn(II) availability. Alternatively, Hst5 may enter the cytoplasm (dotted arrow), bind intracellular Zn(II), and suppress intracellular Zn(II) availability.

To determine whether Hst5 contributes to Zn(II)-dependent nutritional immunity against streptococci, either by promoting Zn(II) starvation or Zn(II) poisoning, we examined the effects of Hst5 on transcription of Zn(II)-responsive genes. *S. pyogenes* (Group A *Streptococcus* or GAS) was used as a model *Streptococcus*, since the transcriptional responses of this species to varying Zn(II) availability is understood (Figure 3), mutant strains lacking key Zn(II) transport proteins are available in our laboratory, and the phenotypes of these mutant strains are known.^48^

**Figure 4:**
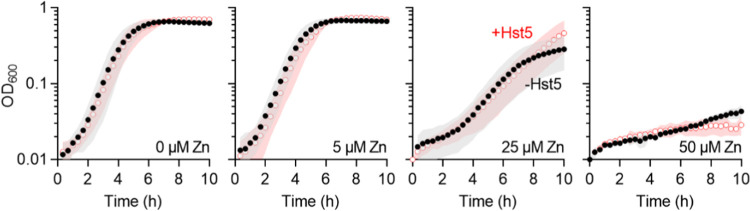
Effects of Zn(II) and Hst5 on growth of GAS. Bacteria (*N* = 3) were cultured in CDM in the presence of Zn(II) (0, 5, 25, or 50 μM), with (○) or without (●) Hst5 (50 μM), and sampled every 20 min for a total of 10 h. While addition of Zn(II) inhibited bacterial growth (*P* = 1.0, <0.0001, and <0.0001 for 5, 25, and 50 μM Zn(II), respectively), addition of Hst5 did not influence this effect (*P* = 0.88, 0.82, 0.83, and 0.56 for 0, 5, 25, and 50 μM Zn(II), respectively).

In response to decreases in Zn(II) availability and Zn(II) starvation, GAS upregulates transcription of the AdcR regulon, including *adcAI* and *adcAII*. Conversely, in response to increases in Zn(II) availability and Zn(II) poisoning, GAS upregulates transcription of the GczA regulon, including *czcD.* Expression of *adcAI*, *adcAII*, and *czcD*, with and without Hst5, was thus examined here. However, poor RNA yields were obtained from the static (nongrowing) bacterial suspensions used in the kill assays. As an alternative approach, GAS was grown in a metal-deplete (low nanomolar concentrations of Zn(II) are routinely detected by ICP MS), chemically defined medium (CDM).^51^ GAS displayed the same phenotypes in CDM and in artificial saliva buffer, *i.e.*, addition of up to 50 μM Hst5 did not affect the growth of this streptococcus and addition of Zn(II) did not influence this outcome (Figure 4), thus validating the approach.

**Figure 5:**
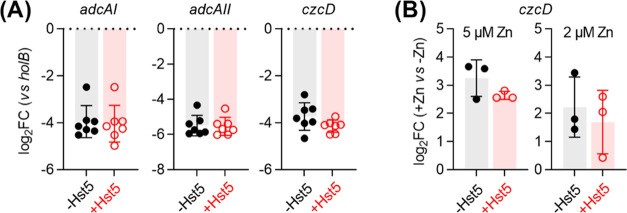
Effects of Hst5 on expression of Zn(II)-responsive genes in GAS. (A) Background expression of all genes. Bacteria (*N* = 7) were cultured in CDM with (○) or without (●) Hst5 (50 μM). Levels of *adcAI*, *adcAII*, and *czcD* mRNA were determined by quantitative real-time polymerase chain reaction (qRT-PCR) and normalized to *holB*. Addition of Hst5 did not affect the background expression of any of the three genes (*P* = 0.35, 0.74, and 0.08 for *adcAI*, *adcAII*, and *czcD*, respectively). (B) Zn(II)-dependent expression of *czcD*. Bacteria (*N* = 3) were cultured in CDM with or without added Zn(II) (2 or 5 μM), with (○) or without (●) Hst5 (50 μM). Levels of *czcD* mRNA were measured by qRT-PCR, normalized to *holB*, and compared with normalized mRNA levels of the corresponding untreated controls (0 μM added Zn(II)). Addition of Hst5 did not affect Zn(II)-dependent expression of *czcD* (*P* = 0.21 and 0.71 for 2 and 5 μM Zn(II), respectively).

In the control experiment, adding Zn(II) alone did not perturb transcription of *adcAI* and *adcAII* in wild-type GAS, but it did induce expression of *czcD* (Figure S3A), consistent with an increase in cellular Zn(II) availability or Zn(II) poisoning. Conversely, adding the Zn(II) chelator TPEN induced expression of *adcAI* and *adcAII*, consistent with a decrease in cellular Zn(II) availability or Zn(II) starvation, but it did not perturb transcription of *czcD* (Figure S3B). By contrast, adding Hst5 perturbed neither the basal expression of *adcAI* or *adcAII* (Figure 5A) nor the Zn(II)-dependent expression of *czcD* (Figure 5B). These results indicate that Hst5 promotes neither Zn(II) starvation nor Zn(II) poisoning to GAS and that Hst5 does not contribute to Zn(II)-dependent nutritional immunity against GAS.

### Hst5 Weakly Suppresses Zn(II) Toxicity

To further explore the hypothesized role of Hst5 in modulating Zn(II) availability, the effects of Hst5 were examined using GAS Δ*adcAI* and Δ*czcD* mutant strains that are deficient in Zn(II) uptake and Zn(II) efflux, respectively (Figure 3). These mutant strains were validated to be sensitive to growth inhibition by the Zn(II) chelator TPEN^52,53^ and added Zn(II),^50,53^ respectively (Figure S4). Although additional Zn(II)-binding lipoproteins such as AdcAII contribute to Zn(II) uptake, AdcAI is thought to act as the primary Zn(II) uptake lipoprotein.^52,53^ Therefore, only the Δ*adcAI* mutant was employed here.

**Figure 6:**
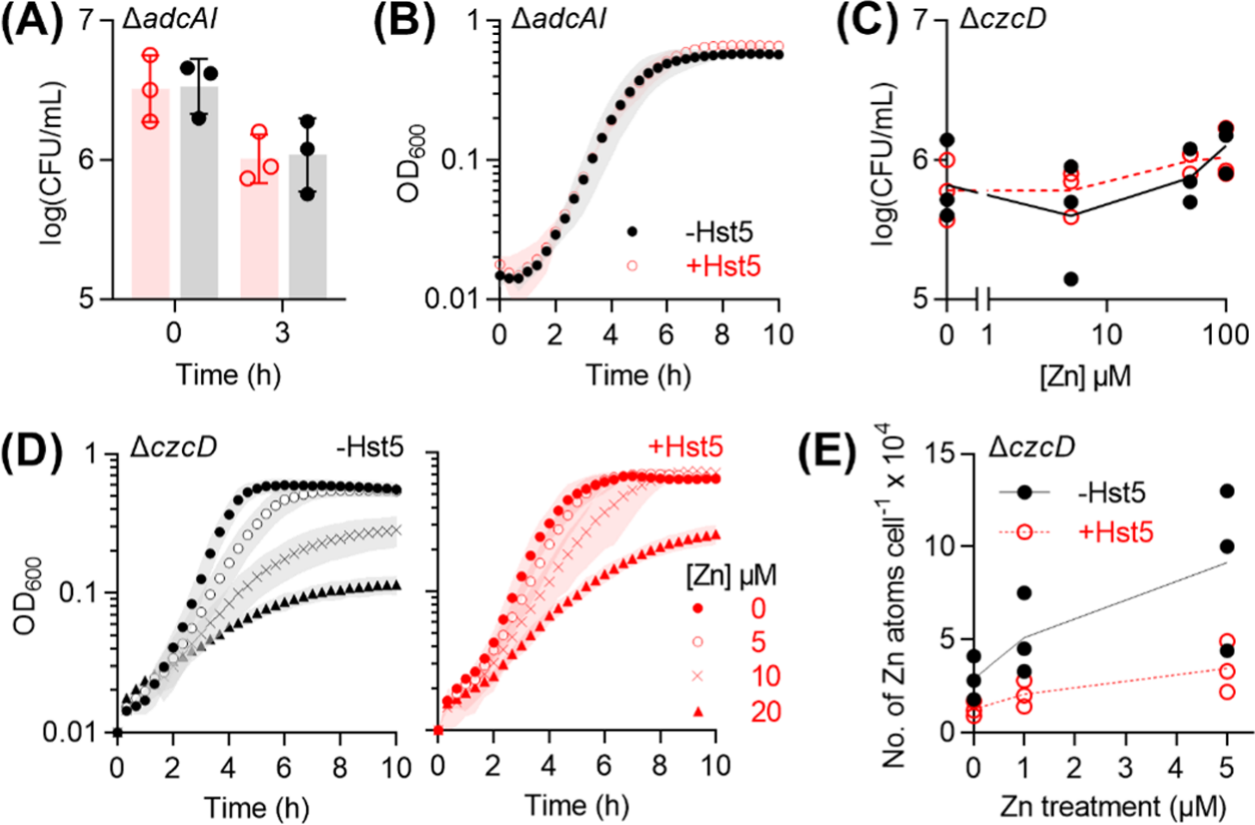
Effects of Hst5 on Zn(II) availability. (A) Survival of Δ*adcAI*. Bacteria (*N* = 3) were incubated in artificial saliva buffer, with (○) or without (●) Hst5 (50 μM), and sampled at *t* = 0 and 3 h for enumeration. Hst5 did not affect the time-dependent survival of the Δ*adcAI* mutant (*P* = 0.90). (B) Growth of Δ*adcAI*. Bacteria (*N* = 2) were cultured in CDM with or without Hst5 (50 μM). Hst5 did not affect the growth of the Δ*adcAI* mutant (*P* = 0.26). (C) Survival of Δ*czcD*. Bacteria (*N* = 3) were incubated in artificial saliva buffer, with or without added Zn(II) (0, 5, 50, or 100 μM), with (○) or without (●) Hst5 (50 μM). Hst5 did not affect the Zn(II)-dependent survival of the Δ*czcD* mutant (*P* value for the interaction between Hst5 and Zn(II) = 0.73). (D) Growth of Δ*czcD*. Bacteria (*N* = 3) were cultured in CDM in the presence of Zn(II) (0–20 μM), with (○) or without (●) Hst5 (50 μM). Hst5 did not affect the growth of the Δ*czcD* mutant in the absence of Zn(II) (*P* = 0.61) but it did affect growth in the presence of Zn(II) (*P* = 0.07, 0.02, and 0.01 for 5, 10, and 20 μM Zn(II), respectively). (E) Levels of cell-associated Zn(II) in Δ*czcD*. Bacteria (*N* = 3) were cultured in CDM in the presence of Zn(II) (0–5 μM), with (○) or without (●) Hst5 (50 μM), and sampled at *t* = 4 h. Levels of cell-associated Zn(II) were measured by ICP MS and normalized to colony counts. Addition of Hst5 had a negative effect on cellular Zn(II) levels (*P* = 0.005).

The Δ*adcAI* mutant strain displayed wild-type survival and growth phenotypes in the presence of Hst5 (Figure 6A,B), strengthening our proposal that Hst5 does not starve GAS of nutrient Zn(II). Similarly, the Δ*czcD* mutant strain displayed wild-type survival phenotype (Figure 6C). However, mild differences between the Δ*czcD* mutant and wild-type strains were observed in growth experiments. While Hst5 did not influence the growth of Zn(II)-treated wild-type organism (see Figure 4), Hst5 weakly but reproducibly improved the growth of the Zn(II)-treated Δ*czcD* mutant strain (Figure 6D). This effect was observed most clearly upon comparing final culture densities after 10 h of growth since the exponential growth rates were unaffected (Figure S5). This growth-promoting effect of Hst5 appeared to require the predicted Zn(II)-binding ligands His15, His18, and His19^54,55^ since the ΔH15,18,19 variant of Hst5 did not rescue the growth of the Zn(II)-treated Δ*czcD* mutant strain (Figure S6, see Table 1 for peptide sequences). These results suggest that Hst5 binds to Zn(II) and suppresses (instead of enhances) the toxicity of an excess of this metal ion to GAS.

Two mechanisms are plausible (see Figure 3): (i) Hst5 binds extracellular Zn(II) and suppresses accumulation of this metal ion in the cytoplasm, leading to less Zn(II) toxicity, or (ii) Hst5 binds cellular Zn(II) and enables more Zn(II) to accumulate in the cytoplasm, but with less toxicity. To distinguish between these models, total cell-associated Zn(II) levels in the Δ*czcD* mutant strain were assessed by ICP MS. Only up to 5 μM Zn(II) was used, since adding 10 μM Zn(II) or more into the cultures inhibited the growth of the Δ*czcD* mutant and did not produce sufficient biomass for metal analyses. Only wild-type Hst5 peptide was used, owing to the large culture volumes required and the high cost of peptide synthesis. Figure 6E shows that Zn(II) treatment increased cell-associated Zn(II) levels in the Δ*czcD* mutant, but co-treatment with Hst5 suppressed this effect. These results are consistent with model (i) above, in which Hst5 binds extracellular Zn(II) and suppresses accumulation of Zn(II) in GAS.

### Hst5 Binds Zn(II) with Micromolar Affinities

**Figure 7:**
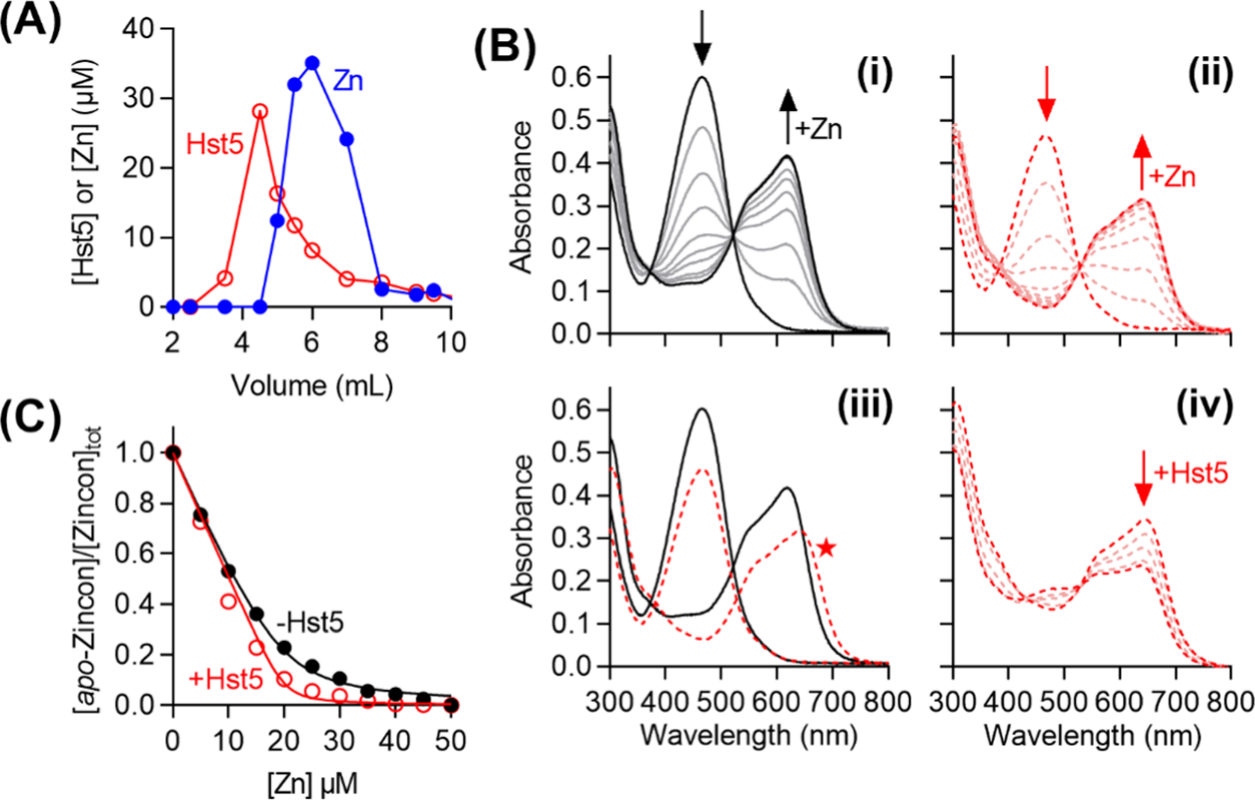
Zn(II) affinity of Hst5. (A) Separation of Hst5 (○) and Zn(II) (●) on a polyacrylamide desalting column. (B) Representative spectral changes upon addition of Zn(II) (0–50 μM) into *apo*-Zincon (20 μM): (i) in the absence (solid traces) or (ii) presence (dashed traces) of Hst5 (20 μM). (iii) Overlaid spectra for 0 and 50 μM Zn(II) from panels (i) and (ii). The new peak at 650 nm is indicated with a star. (iv) Representative spectral changes upon addition of excess Hst5 (0–200 μM) into a solution of Zn(II) (20 μM) and *apo*-Zincon (25 μM). (C) Normalized plot of the absorbance intensities of *apo*-Zincon at 467 nm upon addition of Zn(II), in the absence (●) or presence (○) of Hst5 (20 μM).

To understand how Hst5 weakly modulates Zn(II) availability to GAS and suppresses the toxicity of excess Zn(II) without promoting nutrient Zn(II) starvation, we examined the ability of this peptide to bind Zn(II). Hst5 is thought to bind up to three Zn(II) ions. Previous measurements by isothermal titration calorimetry (ITC) yielded log *K*_Zn(II)_ values of 5.1, 5.0, and 4.0,^56^ indicating that each Zn(II) ion binds to Hst5 with a high micromolar affinity. In agreement with this proposal, a high micromolar concentration of the Zn(II)–Hst5 complex readily dissociated upon passage through a desalting column (Figure 7A). The affinities of Hst5 to Zn(II) were further re-examined here by competing the peptide with the colorimetric Zn(II) indicator Zincon (log *K*_Zn(II)_ ∼ 6.0) in (Mops) buffer and by monitoring solution absorbances of *apo*-Zincon (466 nm) and Zn(II)-Zincon (620 nm) (Figure 7B). The competition curve (in the presence of Hst5) was nearly indistinguishable from the control (in the absence of Hst5) (Figure 7C). Moreover, a new peak at 650 nm appeared in the presence of Hst5 (Figure 7B(iii)), indicating the formation of a new species, likely a ternary complex between Hst5, Zincon, and Zn(II). This peak did not disappear upon adding excess Hst5 (10 molar equiv; Figure 7B(iv)). These results indicate that Hst5 does not compete effectively with Zincon and that this peptide binds Zn(II) with high micromolar affinities, as previously estimated by ITC.^56^

The lack of competition between Hst5 and Zincon as shown in Figure 7 contrasts with a previous study showing an effective competition between Hst5 and Zincon in phosphate buffer, with Hst5 removing 2 molar equiv of Zn(II) from Zincon.^20^ Here it is important to highlight that phosphate binds to Zn(II). Although the affinity of phosphate to Zn(II) is relatively low (log *K*_Zn(II)_ ∼ 2.4),^57^ when used at millimolar concentrations, phosphate can interfere with Zn(II) binding studies by competing for Zn(II). Addition of Zn(II) to *apo*-Zincon in phosphate buffer (50 mM) instead of Mops buffer led to incomplete formation of Zn(II)-Zincon (monitored at 620 nm), suggesting that Zn(II) partitioned between Zincon and phosphate (Figure S7A,B). Conversely, prolonged incubation (>10 min) of a pre-formed Zn(II)-Zincon complex in phosphate buffer led to a loss of the characteristic blue color (Figure S7C), indicating removal of Zn(II) from Zn(II)-Zincon by phosphate alone (without adding Hst5). Therefore, our studies of Zn(II) binding by Hst5 in Mops buffer are likely to be more reliable.

### AdcAI from GAS Binds Zn(II) with Sub-Nanomolar Affinity

**Figure 8:**
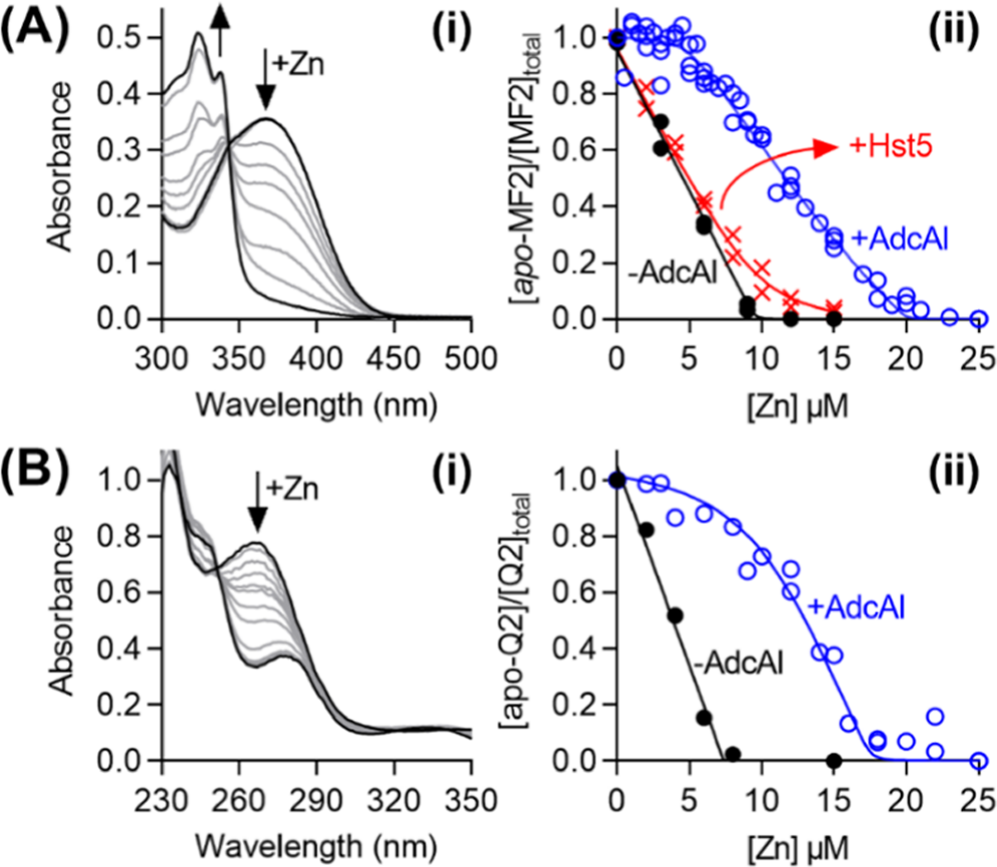
Zn(II) affinity of AdcAI. (A) Low-affinity site. (i) Representative spectral changes upon titration of Zn(II) (0–25 μM) into a mixture of *apo*-Mf2 (10 μM) and AdcAI (5 μM). (ii) Normalized plot of the absorbance intensities of *apo*-MF2 (10 μM) at 377 nm upon addition of Zn(II), in the absence (●) or presence (○) of AdcAI (5 μM). Competition with Hst5 (X; 10 μM) is shown for comparison. (B) High-affinity site. (i) Representative spectral changes upon titration of Zn(II) (0–25 μM) into a mixture of *apo*-Q2 (7.5 μM) and AdcAI (10 μM). (ii) Normalized plot of the absorbance intensities of *apo*-Q2 (7.5 μM) at 262 nm upon addition of Zn(II), in the absence (●) or presence (○) of AdcAI (10 μM).

The low affinity of Hst5 to Zn(II) was clearly insufficient to starve wild-type GAS of nutrient Zn(II) (see Figure 5A), indicating that this peptide does not compete with the high-affinity, Zn(II)-specific uptake protein AdcAI (see Figure 3). Therefore, the Zn(II) affinities of AdcAI were examined here by competition with the colorimetric Zn(II) indicator Mag-fura2 (Mf2). The competition curve, generated by monitoring the solution absorbance of *apo*-Mf2 at 377 nm (Figure 8A(i)), clearly showed two Zn(II) binding sites in AdcAI as anticipated.^58^ The high-affinity Zn(II) binding site outcompeted Mf2, as evidenced by the lack of spectral changes upon adding up to 1 molar equiv of Zn(II) *vs* AdcAI (Figure 8A(ii)). The low-affinity site competed effectively with Mf2 with a log *K*_Zn(II)_ = 8.5 (±0.2). The high-affinity site was better estimated using Quin-2 (Q2) as a competitor. By monitoring the absorbance of *apo-*Q2 at 266 nm, log *K*_Zn(II)_ = 12.5 (±0.2) was obtained for this site (Figure 8B).

The log *K*_Zn(II)_ values for AdcAI determined here were each ∼1000-fold higher than those determined previously by ITC.^58^ ITC can underestimate high metal binding affinities due to lack of sensitivity.^59^ Crucially, Hst5 did not compete with Mf2 for Zn(II) (Figure 8A(ii)). Thus, the relative affinities between Hst5 and AdcAI, determined using the same approach under the same conditions, support the hypothesis that Hst5 does not compete with AdcAI for binding Zn(II). These relative affinities also provide a molecular explanation for why Hst5 does not suppress the availability of nutrient Zn(II) to wild-type GAS.

Hst5 did not affect the growth of GAS even when AdcAI was deleted by mutagenesis (see Figure 6A,B), suggesting that this peptide does not compete with other high-affinity Zn(II) uptake proteins such as AdcAII (see Figure 3). AdcAII was also expressed here for measurements of Zn(II) affinity. However, consistent with a previous report,^60^ recombinant AdcAII co-purified with 1 molar equiv of bound Zn(II), which could not be removed without denaturing the protein. Nevertheless, the reported apparent affinity of the *S. pneumoniae* homologue to Zn(II) (log *K*_Zn(II)_ = 7.7; 67% identity, 81% similarity), determined *via* competition with Mf2,^61^ is ∼100-fold higher than that of Hst5, consistent with our proposal that Hst5 does not compete effectively with AdcAII for binding Zn(II).

## Discussion

### Role of Histatins as Salivary Antimicrobial Agents

The oral cavity is rich in saliva, and interactions between with the components of this host fluid are key for colonization, maintenance, infection, and subsequent transmission of streptococci.^62−64^ For example, exposure to saliva promotes aggregation of some streptococci and blocks adherence to mucosal epithelia.^65,66^ Saliva also contains polysaccharides and glycoproteins that may serve as sources of nutrients. Finally, antimicrobial peptides and enzymes such as lysozyme, lactoperoxidase, and chitinase directly inhibit or kill streptococci.^67^

Given the widely reported antimicrobial activity of Hst5, histatins are thought to function as salivary antimicrobial peptides. Yet, our work shows that Hst5 does not kill seven oral and oropharyngeal streptococcal species under *in vitro* experimental conditions that are characteristic of saliva. It is tempting to speculate that histatins help shape the microbial composition in the healthy oral cavity by suppressing the viability of some microbes (*e.g.*, *C. albicans*) but not others (*e.g.*, streptococci). Future work should carefully examine this potential for histatins to exert a selective antimicrobial activity, to verify that it is not associated only with low ionic strength conditions that are not characteristic of saliva. For example, our work showed that antimicrobial activity of Hst5 against *C. albicans* and *P. aeruginosa* disappears when examined in our artificial saliva buffer (see Figure S2).

To date, there is no consensus as to whether histatin levels in saliva correlate with infection levels in the oral cavity. Comparisons of children or adult patients with and without dental caries have found no variation in salivary histatin levels,^68,69^ higher salivary histatin levels in patients with caries,^70,71^ and lower salivary histatin levels in patients with caries.^72−74^ Similarly, there is no clear correlation between histatin levels and the prevalence of oral *C. albicans* in healthy people^75^ but high histatin levels do correlate with high prevalence of oral candidiasis in immunocompromised patients.^76^ It is important to note that distinct ecological niches exist within the oral cavity. These niches differ in, among many variables, nutrient content, pH, and oxygen tension. Our work does not discount the possibility that histatins exert strong and selective antimicrobial activity in some niches.

### Interactions between Zn(II) and Histatins

Systems for the uptake and efflux of metals such as Zn(II) are important for the survival of streptococci in the oral cavity and oropharynx since salivary concentrations of metals can fluctuate, for example, during and between meals, disease, or human hygiene and dental interventions. In addition, salivary components such as lactoferrin and calprotectin sequester metals and restrict microbial growth.

Our work showed that Hst5 does not contribute to Zn(II)-dependent nutritional immunity against streptococci, since this peptide neither starves our model *Streptococcus* (*S. pyogenes* or GAS) of nutrient Zn(II) nor enhances Zn(II) toxicity to this bacterium. These findings are consistent with results from a genome-wide screen of a GAS mutant library, which did not identify genes involved in Zn(II) uptake or Zn(II) efflux as essential for growth in saliva.^77^ Given the general conservation of Zn(II) homeostasis mechanisms among the streptococci, we anticipate that Hst5 does not contribute to Zn(II)-dependent nutritional immunity against other streptococci.

The low affinity of Hst5 to Zn(II), particularly compared with the high affinities of the Zn(II) uptake lipoproteins AdcAI and AdcAII, explains why Hst5 does not starve GAS (and, presumably, other streptococci) of nutrient Zn(II). Here, the antimicrobial protein calprotectin provides a useful comparison. Calprotectin binds two Zn(II) ions with affinities (log *K*_Zn(II)_ > 11 and >9.6)^78^ that are comparable to those of AdcAI and higher than that of AdcAII. Indeed, adding calprotectin induces a robust Zn(II) starvation response in streptococci,^79,80^ consistent with its established role in nutritional immunity.

Its low affinity to Zn(II) also explains why Hst5 only weakly suppresses the availability of excess (toxic) Zn(II) to GAS *in vitro*. Like most culture media, our CDM^51^ contains phosphate (∼6 mM) and amino acids (∼6 mM total), which would outcompete Hst5 (50 μM) for binding Zn(II).^57^ However, if these competing ligands become depleted, for example as a result of bacterial growth, then Hst5 may become competitive and bind Zn(II), particularly when Zn(II) concentrations are high. Such shifts in Zn(II) speciation likely explain why the protective effect of Hst5 on the GAS Δ*czcD* mutant strain during conditions of Zn(II) stress became apparent only at the later stages of growth (see Figure S5). The increased binding of Zn(II) to Hst5 in these later stages of growth may suppress nonspecific Zn(II) import into the GAS cytoplasm, for instance by outcompeting promiscuous divalent metal transporters.

Unlike *in vitro* growth media, saliva and its components are continuously replenished *in vivo*. Saliva contains ∼10 mM phosphate^81,82^ and proteinaceous components that also bind Zn(II).^83^ Thus, *in vivo*, Hst5 is unlikely to be competitive for binding Zn(II). Nonetheless, synergistic effects between Zn(II) and Hst5 may occur *in vivo*, but likely *via* indirect mechanisms that do not rely on direct binding of Zn(II) to Hst5 and formation of a Zn(II)–Hst5 complex. Zn(II) and Hst5 may separately target the same cellular pathways in a microbe, leading to the enhancement of the antimicrobial activity of Hst5 by Zn(II). Alternatively, Zn(II) may disable cellular pathways that render the target microbe more susceptible to the separate action of Hst5 on a different cellular pathway (or *vice versa*), again leading to the enhancement of microbial killing. Indirect interactions between Zn(II) and Hst5 may also exert subtle effects on microbial physiology that do not lead to a direct antimicrobial action and thus are not captured by the assays described here. For example, a combination of Zn(II) and Hst5 at nonlethal doses is thought to reduce the virulence of *C. albicans*.^84^ Whether Hst5 reduces the virulence of streptococci and subsequently enables these organisms to become the dominant commensal microorganisms in the oral cavity and oropharynx is an intriguing concept that warrants further investigation.

## Methods

### Data Presentation

Except growth curves, individual replicates from microbiological experiments are plotted, with shaded columns representing the means and error bars representing standard deviations. Growth curves show the means, with shaded regions representing standard deviations. The number of biological replicates (independent experiments, using different starter cultures and different medium or buffer preparations, performed on different days; *N*) is stated in figure legends. In the case of metal–protein and metal–peptide titrations, individual data points from two technical replicates (independent experiments performed on different days but using the same protein or peptide preparation) are plotted, but only representative spectra are shown for clarity of presentation.

### Statistical Analyses

Descriptive statistics are displayed on all graphical plots. Inferential statistics have been computed for all data and the relevant *P* values are listed in figure legends. Unless otherwise stated, tests of significance used two-way analysis of variance using the statistical package in GraphPad Prism 8.0. All analyses were corrected for multiple comparisons.

### Reagents

The nitrate salt of Zn(II) was used in experiments. Numerous additional tests did not identify any observable difference in the results when the chloride or sulfate salts of Zn(II) were used. Peptides were synthesized commercially with free N- and C-termini as the acetate salt, purified to >95% (GenScript), and confirmed to be metal-free by ICP MS. Concentrations of stock peptide solutions were estimated using solution absorbances at 280 nm in Mops buffer (50 mM, pH 7.4; ε_280_ = 2667 cm^–1^). Concentrations of fluorometric and colorimetric metal indicators (Zincon, PAR, Mf2, Q2) were standardized using a commercial standard solution of copper chloride. Concentrations of optically silent chelators (NTA) were standardized by competition with a standardized solution of Zn(II)-Zincon.

### Strains and Culture Conditions

All bacterial strains (Table S1B) were propagated from frozen glycerol stocks onto solid THY (Todd Hewitt + 0.2% yeast extract) medium without any antibiotics and incubated overnight in the presence of 5% v/v of atmospheric CO_2_. Liquid cultures were prepared in THY or CDM.^51^ All solid and liquid growth media contained catalase (50 μg/mL).

### Streptococcal Kill Assays

Fresh colonies from an overnight THY agar were resuspended to 10^6^–10^7^ CFU/mL in either potassium phosphate buffer (10 mM, pH 7.4) or artificial saliva buffer (pH 7.2; Table S1A). The cultures were incubated at 37 °C with or without Hst5 and/or Zn(II) as required. At *t* = 0 and 3 h, cultures were sampled and serially diluted in CDM. Exactly 10 μL of each serial dilution was spotted onto THY agar. Colonies were enumerated after overnight incubation at 37 °C.

### *C. albicans* Kill Assays

Cells from a fresh YPD plate were harvested, washed three times in phosphate-buffered saline (PBS), and resuspended in either potassium phosphate buffer (10 mM, pH 7.4) or saliva salts (pH 7.2) to an OD_600_ of 0.4 (∼5 × 10^6^ CFU/mL). Cultures were incubated with or without Hst5 at 37 °C. Tubes were inverted every 20 min to maintain cell suspension. At *t* = 0, 1, and 3 h, samples were taken, serially diluted, and plated onto YPD agar. Colonies were numerated following overnight incubation at 30 °C.

### Growth Assays

Colonies from an overnight THY agar were resuspended in CDM to an OD_600_ = 0.02 and dispensed into wells in flat-bottomed 96-well plates (200 μL per well) containing Hst5 and/or Zn(II) as required. Bacterial growth was monitored using an automated microplate shaker and reader. Each plate was sealed with a gas-permeable, optically clear membrane (Diversified Biotech). OD_600_ values were measured every 20 min for 10 h. The plates were shaken immediately before each reading (200 rpm, 1 min, double-orbital mode). OD_600_ values were not corrected for path length (*ca*. 0.58 cm for a 200 μL culture).

### RNA Extraction

Colonies from an overnight THY agar were resuspended in CDM to an OD_600_ = 0.02 and incubated in 24-well plates (1.6 mL per well), with or without Hst5 or Zn(II) as required, without shaking, at 37 °C. Each plate was sealed with a gas-permeable, optically clear membrane (Diversified Biotech). At *t* = 4 h, cultures were centrifuged (4000*g*, 4 °C, 5 min) and the resulting bacterial pellets were resuspended immediately in RNAPro Solution (0.5 mL; MP Biomedicals). Bacteria were lysed in Lysing Matrix B and total RNA was extracted following the manufacturer’s protocol (MP Biomedicals). Crude RNA extracts were treated with RNase-Free DNase I (New England Biolabs). Removal of gDNA was confirmed by PCR using gapA-check-F/R primers (Table S1C). gDNA-free RNA was purified using Monarch RNA Clean-up Kit (New England Biolabs) and visualized on an agarose gel.

### qRT-PCR Analyses

cDNA was generated from RNA (1.6 μg) using SuperScript IV First-Strand Synthesis System (Invitrogen). Each qRT-PCR reaction (20 μL) contained cDNA (5 ng) as template and the appropriate primer pairs (0.4 μM; Table S1C). Samples were analyzed in technical duplicates. Amplicons were detected with Luna Universal qRT-PCR Master Mix (New England Biolabs) in a CFXConnect Real-Time PCR Instrument (Bio-Rad Laboratories). *C*_q_ values were calculated using LinRegPCR^85^ after correcting for amplicon efficiency. *C*_q_ values of technical duplicates were typically within ±0.25 of each other. *holB*, which encodes DNA polymerase III, was used as reference gene. Its transcription levels were verified to remain constant in the experimental conditions tested here.

### Cellular Metal Content

Colonies from an overnight THY agar were resuspended in CDM to an OD_600_ = 0.02 and incubated at 37 °C with or without Hst5 and/or Zn(II) as required. At *t* = 4 h, an aliquot was collected for the measurement of plating efficiency (colony counts). The remaining cultures were centrifuged (5000*g*, 4 °C, 10 min). The resulting bacterial pellets were washed once with ice-cold wash buffer (1 M d-sorbitol, 50 mM Tris–HCl, 10 mM MgCl_2_, 1 mM ethylenediaminetetraacetic acid (EDTA), pH 7.4) and twice with ice-cold PBS. The final pellets were dissolved in concentrated nitric acid (100 μL), heated (85 °C, 1.5 h), and diluted to 3.5 mL with 2% nitric acid. Total metal levels were determined by ICP MS and normalized to colony counts.

### Elution of Zn(II)–Hst5 on a Desalting Column

*Apo*-Hst5 (100 μM) was incubated with 1.5 molar equiv of Zn(II) for 15 min at the bench and loaded onto a polyacrylamide desalting column (1.8 kDa molecular weight cutoff, Thermo Scientific). Peptide and Zn(II) were eluted from the column using Mops buffer (50 mM, pH 7.4). The concentration of Hst5 in each fraction was determined using QuantiPro BCA Assay Kit (Merck) and known quantities of Hst5 as standards. The concentration of Zn(II) was determined using the colorimetric Zn(II) ligand PAR against a standard curve.

### Equilibrium Competition Reactions

Our approach to determine metal-binding affinities followed that described by Young and Xiao.^59^ For each competition (eq 1), a master stock was prepared to contain both competing ligands (L1 and L2) in Mops buffer (50 mM, pH 7.4). Serial dilutions of the metal (M) were prepared separately in deionized water. Exactly 135 μL of the master stock was dispensed into an Eppendorf UVette and 15 μL of the appropriate metal stock was added. Solution absorbances were recorded and used to calculate concentrations of *apo*- and metalated forms of each ligand. These concentrations were plotted against metal concentrations and fitted in DynaFit^86^ using binding models as described in the text. The known association or dissociation constants for all competitor ligands are listed in Table S1D
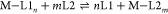
1

### Overexpression and Purification of AdcAI and AdcAII

Nucleic acid sequences encoding the soluble domains of AdcAI (from Thr21) and AdcAII (from Thr31) from M1GAS strain 5448 were subcloned into vector pSAT1-LIC using primers listed in Table S1C. This vector generates N-terminal His6-SUMO fusions with the target proteins. The resulting plasmids were propagated in *Escherichia coli* Dh5α, confirmed by Sanger sequencing, and transformed into *E. coli* BL21 Rosetta 2(DE3).

To express the proteins, transformants were plated onto Lysogeny Broth (LB) agar. Fresh colonies were used to inoculate LB (1 L in 2 L baffled flasks) to an OD_600_ of 0.01. The culture medium contained ampicillin (100 μg/mL) and chloramphenicol (33 μg/mL). Cultures were shaken (200 rpm, 37 °C) until an OD_600_ of 0.6–0.8 was reached, and expression was induced by adding isopropyl β-d-1-thiogalactopyranoside (IPTG) (0.1 mM). After shaking for a further 16 h at 20 °C, the cultures were centrifuged (4000*g*, 4 °C) and the pellets were resuspended in buffer A500 (20 mM Tris–HCl, pH 7.9, 500 mM NaCl, 5 mM imidazole, 10% glycerol).

To purify proteins, bacteria were lysed by sonication (40 kpsi), centrifuged (20,000*g*, 4 °C), and filtered through a 0.45 μm poly(ether sulfone) (PES) membrane filtration unit. Clarified lysates were loaded onto a HisTrap HP column (Cytiva). The column was washed with 10 column volumes (CV) of buffer A500 followed by 10 CV of buffer A100 (20 mM Tris–HCl, pH 7.9, 100 mM NaCl, 10% w/v glycerol) containing imidazole (5 mM). Both AdcAI and AdcAII were bound to the column and subsequently eluted with 3 CV of buffer A100 containing 250 mM imidazole followed by 5 CV of 500 mM imidazole. Protein-containing fractions were loaded onto a Q HP column (Cytiva). The column was washed with 5 CV of buffer A100 and bound proteins were eluted using a step gradient of 0, 10, 15, and 20% buffer C1000 (20 mM Tris–HCl, pH 7.9, 1000 mM NaCl, 10% w/v glycerol). Eluted proteins were incubated overnight at 4 °C with hSENP2 SUMO protease to cleave the His6-SUMO tag from the target protein. Samples were passed through a second Q HP column and the flowthrough fractions containing untagged target protein were collected.

## Supporting Information Available

Recipe for artificial saliva buffer, generated by combining the known salt composition of human saliva (Table S1A); microbial strains used in this study (Table S1B); list of primers used in this study (Table S1C); and list of fluorometric and colourimetric metal indicators used in this study (Table S1C) (XLSX)
Effects of Hst5 on survival of streptococci after 24 h of exposure (Figure S1); effects of Hst5 on survival of control organisms *P. aeruginosa* and *C. albicans* (Figure S2); effects of Zn and TPEN on gene expression in wild-type GAS (Figure S3); characteristic phenotypes of the GAS Δ*adcAI* and Δ*czcD* mutant strains (Figure S4); effects of Hst5 on growth of GAS Δ*czcD* mutant strain (Figure S5); effects of Hst5 and the ΔH15,18,19 variant on the GAS Δ*czcD* mutant strain (Figure S6); and competition between phosphate and Zincon for binding Zn (Figure S7) (PDF)
