# Supplementary material for: Salivary Antimicrobial Peptide Histatin-5 Does Not Display Zn(II)-Dependent or -Independent Activity against Streptococci
Source: ACS Infect Dis. 2023 Feb 24;9(3):631–42. doi: 10.1021/acsinfecdis.2c00578 (PMC10012264; doi:10.1021/acsinfecdis.2c00578)
Supplement: Supplementary file 2 — id2c00578_si_002.pdf [file id2c00578_si_002.pdf]

SUPPORTING INFORMATION

The salivary antimicrobial peptide histatin-5 does not display Zn(II)-dependent or -independent activity against streptococci

Louisa J Stewart[1$], YoungJin Hong[1$], Isabel R Holmes[1], Samantha J Firth[1], Yasmin Ahmed[2], Janet Quinn[2], Yazmin Santos[3], Steven L Cobb[3], Nicholas S Jakubovics[4], Karrera Y Djoko[1*]

[1]Department of Biosciences, Durham University, Durham DH1 3LE, United Kingdom

[2]Biosciences Institute, Newcastle University, Newcastle NE2 4HH, United Kingdom

[3]Department of Chemistry, Durham University, Durham DH1 3LE, United Kingdom

[4]School of Dental Sciences, Newcastle University, Newcastle NE2 4BW, United Kingdom

*Corresponding author

karrera.djoko@durham.ac.uk

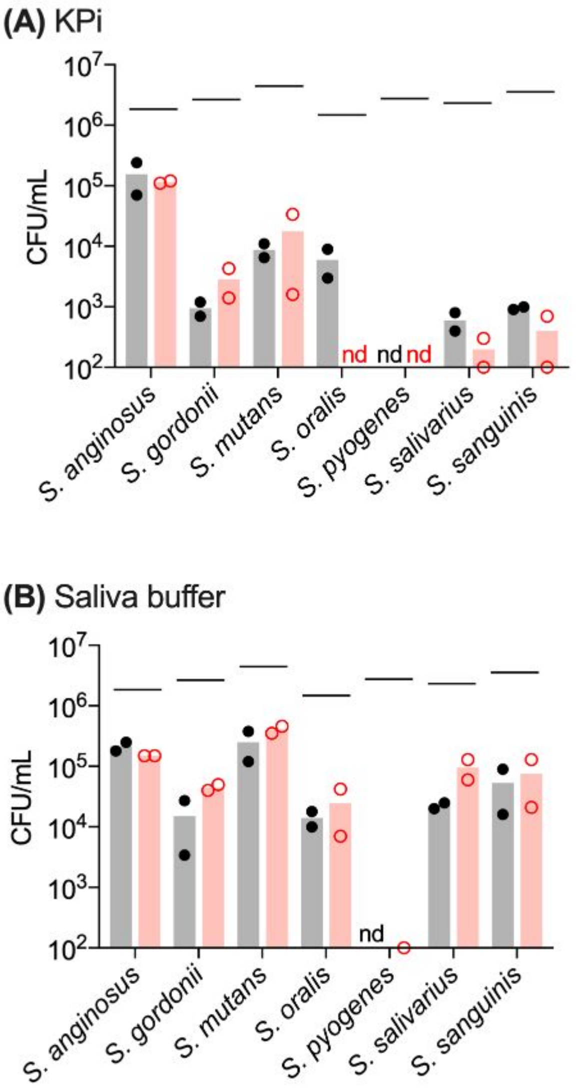

Figure S1. Effects of Hst5 on survival of streptococci after 24 h of exposure. Bacteria were incubated in **(A)** phosphate buffer (10 mM, pH 7.4; N = 2) or **(B)** artificial saliva buffer (pH 7.2-7.4; N = 2), with (○) or without (●) Hst5 (50 μM), and sampled at t = 24 h for enumeration. Bacterial counts in the original inoculum at t = 0 h are shown as horizontal lines. nd, not detected (the detection limit of the assay was 10^2 CFU/mL).

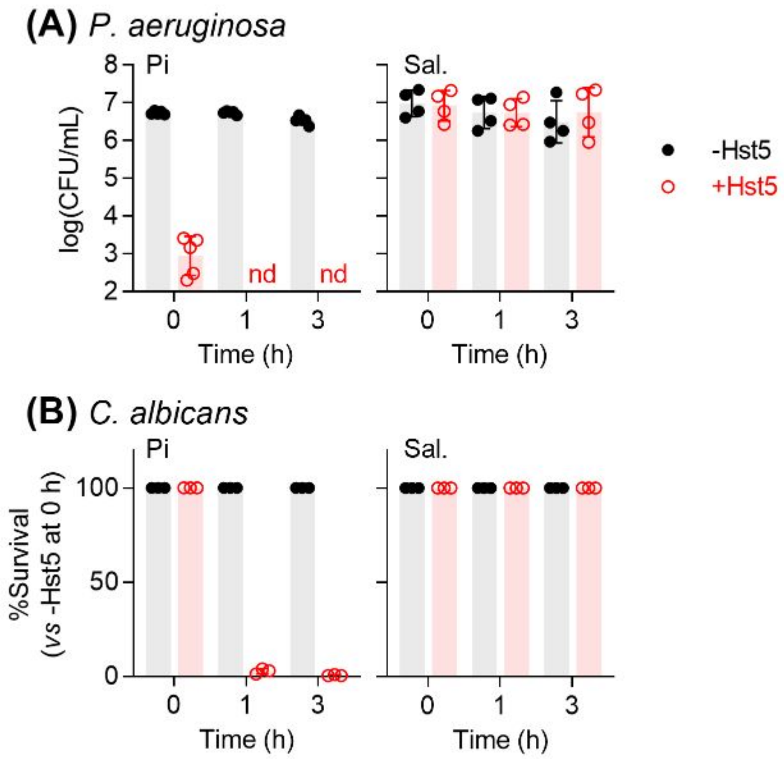

Figure S2. Effects of Hst5 on survival of control organisms (A) ***P. aeruginosa*** **and (B)** ***C. albicans.*** *P. aeruginosa* (~5 x 10^6 CFU/ml, *N* = 5) or *C. albicans* (~5 x 10^6 CFU/ml, *N* = 3) were incubated in potassium phosphate buffer (Pi; 10 mM, pH 7.4) or artificial saliva buffer (Sal.), with (○) or without (●) Hst5 (50 μM), and sampled at t = 0, 1, or 3 h for enumeration. *nd*, not detected (detection limit log(CFU/ml) = 2). Note that at *t* = 0 h, approximately 5 min passed between addition of Hst5 into microbial cultures and plating out for enumeration. Addition of Hst5 had a negative effect on the survival of both organisms in phosphate buffer ($P < 0.0001$ for each organism) but not in artificial saliva buffer ($P = 0.77$ and 1 for *P. aeruginosa* and *C. albicans*, respectively).

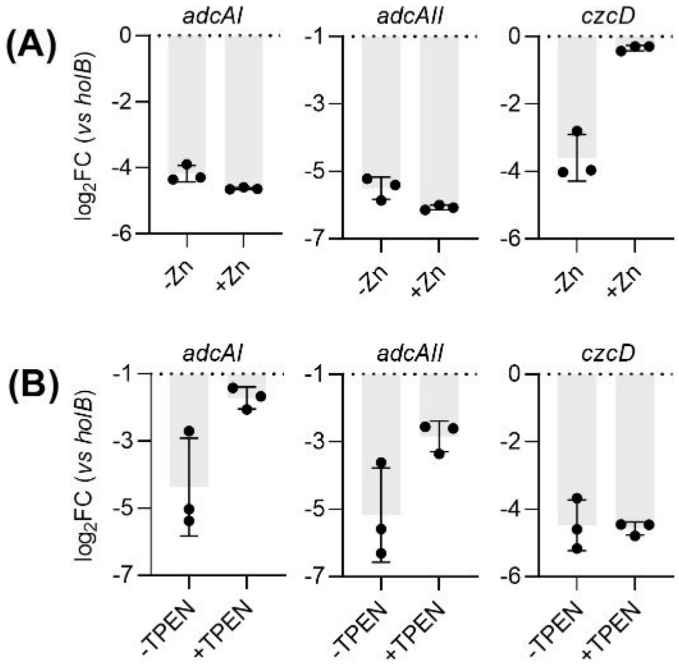

Figure S3. Effects of (A) Zn and (B) TPEN on gene expression in wild-type GAS. Bacteria (*N* = 3) were cultured in CDM-glucose for 4 h with (+) or without (-) added Zn (5 µM) or TPEN (100 nM) as indicated. Levels of *adcAI*, *adcAII*, and *czcD* mRNA were determined by qRT-PCR and normalised to *holB*. Growth in the presence of Zn led to upregulation of *czcD* ($P < 0.0001$) but not *adcAI* ($P = 0.32$) or *adcAII* ($P = 0.15$). Growth in the presence of TPEN led to upregulation of *adcAI* ($P = 0.01$) and *adcAII* ($P = 0.03$) but not *czcD* ($P = 1.0$).

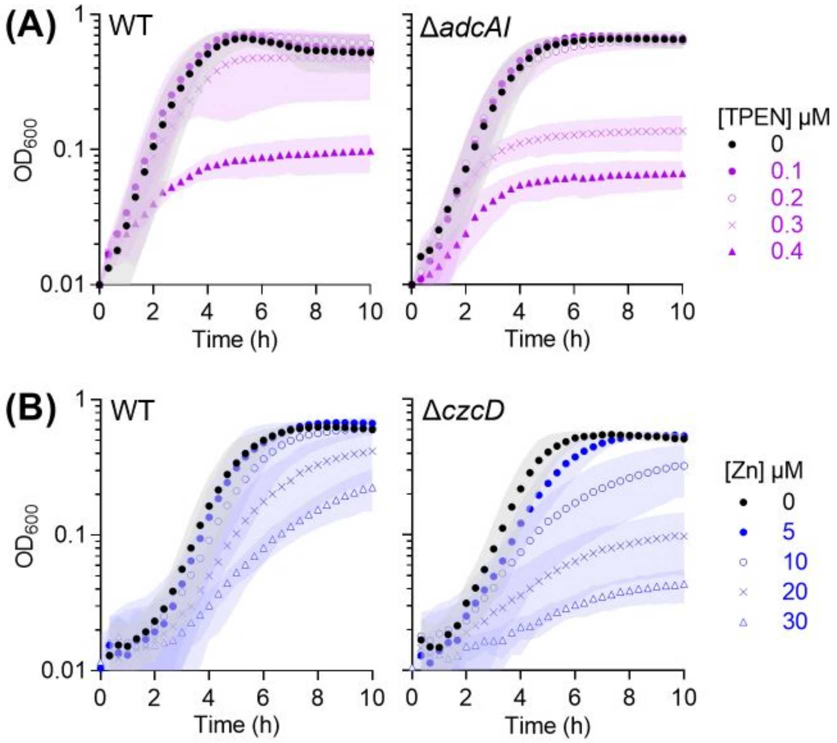

Figure S4. Characteristic phenotypes of the GAS Δ*adcAI* and Δ*czcD* mutant strains. (A) TPEN-sensitive phenotype of the Δ*adcAI* mutant. Bacteria (*N* = 3) were cultured in CDM in the presence of TPEN (0 – 0.4 μM). **(B) Zn-sensitive phenotype of the Δ*czcD* mutant.** Bacteria (*N* = 3) were cultured in CDM in the presence of added Zn (0 – 30 μM).

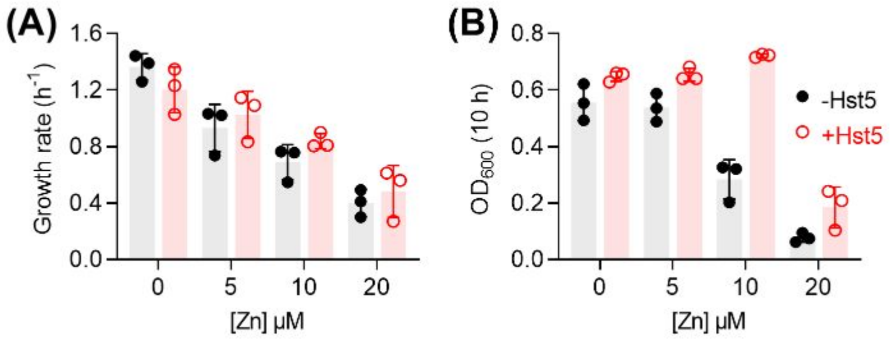

Figure S5. Effects of Hst5 on growth of GAS ∆*czcD* mutant strain. Bacteria (N = 3) were cultured in CDM in the presence of Zn (0 – 20 μM), with (○) or without (●) Hst5 (50 μM). The resulting growth curves (shown in Figure 6D) were used to determine **(A)** exponential growth rates and **(B)** final culture densities. Addition of Hst5 had no effect on growth rates (P = 0.49) but it did have a positive effect on final culture densities (P < 0.0001).

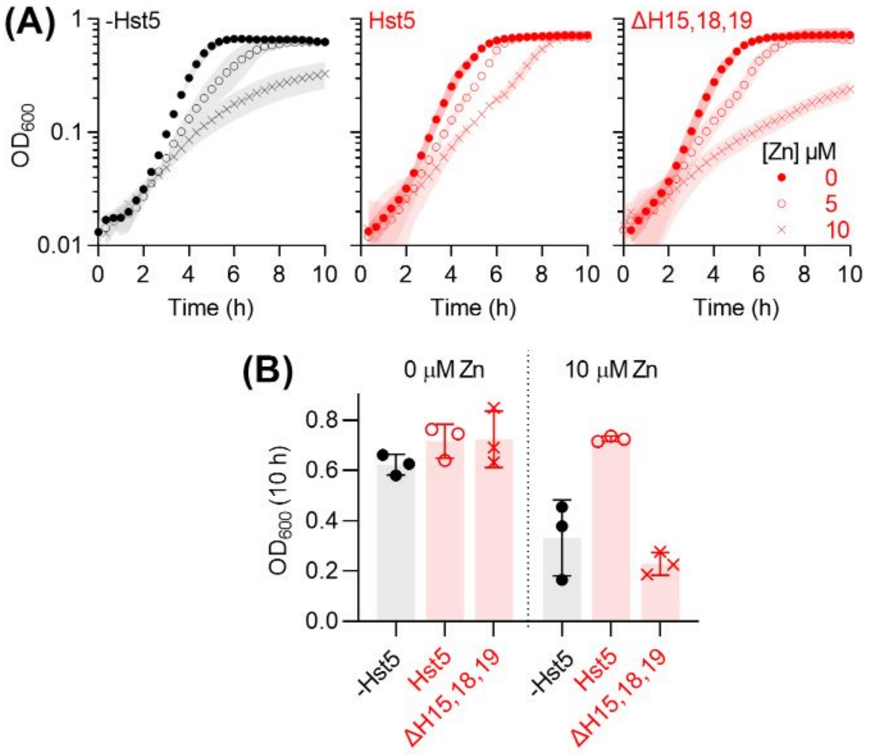

Figure S6. Effects of Hst5 and the ΔH15,18,19 variant on the GAS Δ*czcD* mutant strain. (A) Bacteria (N = 3) were cultured in CDM with added Zn (0, 5, 10 μM), in the absence (-Hst5) or presence of Hst5 or the ΔH15,18,19 variant (50 μM each; see Table 1 for peptide sequences). **(B)** Plot of OD_{600} values at t = 10 h from growth curves in panel A. Hst5 increased culture densities in the presence of 10 μM Zn (P = 0.0002) but the ΔH15,18,19 variant did not (P = 0.27).

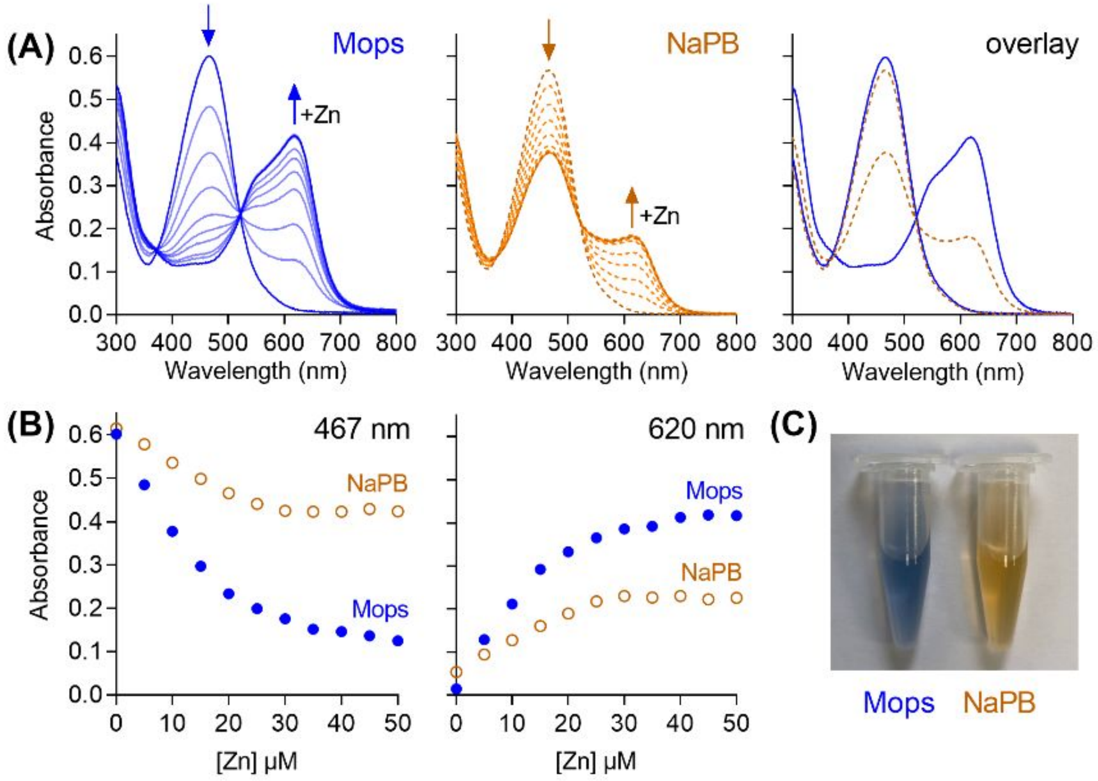

Figure S7. Phosphate buffer competes with Zincon for Zn. (A) Spectral changes upon titration of Zn (0 – 50 μM) into *apo*-Zincon (20 μM) in Mops buffer (50 mM, pH 7.4; solid traces) or sodium phosphate buffer (50 mM, pH 7.4; NaPB, dashed traces). **(B)** Plot of absorbance values at 467 nm or 620 nm from panel A. **(C)** Loss of the characteristic blue colour of Zn-Zincon complex upon incubation in NaPB for >10 min.
